# The minimal invasive direct anterior approach in combination with large heads in total hip arthroplasty - is dislocation still a major issue? a case control study

**DOI:** 10.1186/1471-2474-15-80

**Published:** 2014-03-12

**Authors:** Steffen Hoell, Marius Sander, Georg Gosheger, Helmut Ahrens, Ralf Dieckmann, Gregor Hauschild

**Affiliations:** 1 Center of arthroplasty and revision arthroplasty, Department of General Orthopedics and Tumor Orthopedics, University Hospital Muenster, 48149 Muenster, Germany; 2 University Hospital Muenster, 48149 Muenster, Germany; 3 Department of general orthopedics and tumor orthopedics, University Hospital Muenster, 48149 Muenster, Germany

**Keywords:** Direct anterior approach, Large heads, Minimal invasive surgery, Total hip arthroplasty, Dislocation rate

## Abstract

**Background:**

There have been increasing numbers of publications in recent years on minimally invasive surgery (MIS) for total hip arthroplasty (THA), reporting results with the use of different head sizes, tribologic and functional outcomes. This study presents the results and early complication rates after THA using the direct anterior approach (DAA) in combination with head sizes ≥ 36 mm.

**Methods:**

A total of 113 patients with THA were included in the study. The Harris Hip Score (HHS) was determined, a radiographic evaluation was carried out, and complications were recorded. The minimum follow-up period was 2 years (means 35 ± 7 months).

**Results:**

The HHS improved from 43.6 (± 12) to 88.2 (± 14; *P* < 0.01). One early infection occurred, one periprosthetic fracture, and three cases of aseptic stem loosening. No incorrect positioning of the implants was observed, and there were no dislocations.

**Conclusion:**

THA with the minimally invasive DAA in combination with large heads is associated with good to very good functional results in the majority of cases. The complication rates are not increased. The rate of dislocation mainly as an complication of the first two years can be markedly reduced in particular.

## Background

There have been increasing numbers of publications in recent years on minimally invasive surgery (MIS) for total hip arthroplasty (THA), reporting results with the use of different head sizes and tribologic and functional outcomes. Particularly in the early postoperative period, MIS hip arthroplasty shows advantages in comparison with conventional hip arthroplasty. The hospitalization period is shorter, hip function and gait show shorter recovery times, the range of motion of the hip in the first 12 weeks is markedly increased, and pain is significantly reduced in comparison with the lateral approach [1-3]. In comparison with the minimally invasive anterolateral approach the direct anterior approach (DAA), could show that the abductors in particular are spared [4,5]. In a study comparing the posterolateral and DAA, the DAA was associated with better results in regard to function and pain for up to 3 months postoperatively [6].

To meet the patients’ increasing functional expectations, large-diameter heads are increasingly being used again. When heads with diameters of 38 mm and 44 mm were used, the range of motion in a model was significantly increased in comparison with 28-mm and 32-mm heads [7]. Similar results have been reported in other studies, in which the better head–neck ratio was emphasized as the reason for the improvement [8-12]. In addition, range of motion is improved with large heads, as a result of lower component-to-component impingement [9,10]. The dislocation rate in THA is reported to be in the range of 0.5–5.0% [10,12,13]. Dislocation rates were reduced from 3.1% and 4.45% to 0.8% by the use of heads with diameters of 32 and 36 mm, in comparison with 28-mm heads [14-16]. However, the approach used and the patient’s age and sex also influence dislocation rates [17-19]. The reduced risk of dislocation with larger heads must of course not lead to poorer wear rates. The risk of osteolysis was reduced by 87% using cross-linked polyethylene [20]. No increased wear was observed even in combination with large heads up to 36 mm in size [21].

This study presents the results after THA via the direct anterior approach in combination with head sizes ≥ 36 mm.

### Methods

A total of 107 patients (55 male, 52 female, average age 59.6 ( min. 17 - max .78)) with 113 THAs were included in the study. The inclusion criteria were THA via DAA in combination with a minimum head size of 36 mm (n = 79), 40 mm (n = 28), and 44 mm (n = 6). Exclusion criteria were high hip dislocations with a femoral shortening osteotomy and patients with an indication for hip arthroplasty due to metastatic involvement. The patients’ mean age was 59.6 years (range 17–78). The indication for THA was primary osteoarthritis (n = 73), dysplastic osteoarthritis (n = 12), and secondary osteoarthritis (femoral head necrosis n = 15, post-traumatic osteoarthritis n = 4, and secondary osteoarthritis after Legg–Calvé–Perthes disease or epiphysiolysis capitis femoris, n = 9). All of the cups were cement less (Trident PSL cup, Stryker Orthopedics) and ranged from 46 mm to 64 mm in size. Additional screw fixation of the cup was carried out in seven cases. Fixation of the stems was cementless in 91 cases (Accolade I TMZF, Stryker Orthopedics) and cemented in 22 (MV 40 Stryker Orthopedics). The head material was ceramic in 90 cases and metal in 23. The inlays were all made of sequentially cross-linked X3™ polyethylene, so that use of 36-mm heads was possible starting from a diameter of 46 mm.

The retrospective analysis was carried out using the available patients’ files and radiographic documentation, along with a follow-up examination. The Harris Hip Score (HHS) was calculated preoperatively and at the time of the follow-up examination. Postoperative complications were noted. The cup inclination angle was measured on the a.p. pelvic x -ray. Decentering of the head was measured on the basis of digital radiographic images. Osteolyses and signs of loosening were described using the DeLee and Charnley method [22]. Osteolyses of the stem were measured in the Gruen zones [23]. The minimum follow-up period was 2 years (mean 35 ± 7 months).

All patients included gave their consent in participation in this study.

An Ethics approval was not nescessary proved by the Ethics Committee. Statistical analysis was carried out using the PASW Statistics program, version 18 (SPSS Inc., Chicago, Illinois), with the *t*-test for independent random samples and the significance level set at *P* < 0.05.

## Results

The preoperative HHS was 43.6 (± 12). At the follow-up examination, the mean value was 88.2 (± 14). Significant improvement was achieved (*P* < 0.01). Excellent scores were noted in 66.3% of the patients, with 18.9% in the good range. Only 9.5% had moderate scores. Poor surgical results were noted in 5.3% of the patients.

The female patients achieved a mean of 88 points, while males had 88.4 points at the follow-up (*P* > 0.05). The mean age in the female group was 59.1 years, in comparison with 60.4 in the males. In 25 women, the surgical results were excellent (51%). Overall, 38 of the male patients had excellent results, with over 90 points (82.6%). There were no statistically significant differences in the results between the two groups (*P* = 0.69).

Three groups were formed based on age. Group A was defined as up to age 50 (21%), group B from 51 to 70 years (59%), and group C over 70 (20%). Postoperatively, patients in group A had average scores of 86.9 (± 10). In Group B, the HHS at the follow-up was 89.4 (± 18). In group C, the mean HHS at the follow-up examination was 86.1 (± 14). There were no significant differences among the postoperative scores (*P* > 0.05). The percentages of patients in the three groups with scores over 90 were 55%, 73.2%, and 57.9%, again with no statistically significant differences.

The influence of body mass index (BMI) on the patients’ functional outcome was also analyzed using three groups: group A, BMI < 25 kg/m^2^, group B 25–35 kg/m^2^, and group C > 35 kg/m^2^. At the follow-up examination, the HHS scores were 88.6 (± 13), 88.8 (± 16), and 84.9 (± 12), respectively. There were no significant differences between the groups (*P* > 0.05). The proportions of patients with scores over 90 were 63%, 74.5%, and 38.5%, respectively — again without statistical significance, but with a trend towards better results in the groups with BMI < 35 kg/m^2^.

When the influence of head sizes of 36 mm (n = 79), 40 mm (n = 28), and 44 mm (n = 6) on the HHS at the follow-up examination was analyzed, no significant differences were seen: 88.2 (± 16), 88.4 (±8), and 89.6 (± 10), respectively (*P* > 0.05).

The cup inclination angle measured on anteroposterior radiography was 44.9° (± 5°). No osteolyses around the cups could be found. No cases of head movement due to wear were observed. An increase in osteolyses in Gruen zones 1 and 7 to more than 2 mm was seen in three cases because of aseptic loosening.

### Complications

The dislocation rate was zero. No deep vein thromboses or pulmonary embolisms occurred. Delayed wound healing followed in two cases, which were treated conservatively. Early infection developed in one patient after 10 days, with confirmed *Staphylococcus aureus* (0.9%). A one stage revision was carried out, with debridement, exchange of head and inlay and antibiotic treatment One patient suffered a periprosthetic fracture during the rehabilitation programm. A revision to a modular cementless revision stem by using a lateral approach was carried out (0.9%). The patient had an above-knee amputation on the contralateral side.

Aseptic loosening of the cementless stem occurred in three patients during the first to sixth months after surgery (2.7%).

## Discussion

There has been increasing discussion of MIS for THA in recent years, and it is now regarded as an established procedure. Whereas THA with head diameters of 22 mm were customary in Charnley’s time, the use of larger diameters has been investigated again more recently. Particularly in the early postoperative phase, MIS THA has been shown to have advantages over conventional surgical procedures. The hospitalization period is shorter, hip function and gait show shorter recovery times, the range of movement during the first 12 weeks is markedly increased, and pain symptoms are significantly less than with the lateral approach [1]–[5]. Comparing the minimally invasive anterolateral approach and DAA, it has been shown that the abductors in particular are spared with the DAA [4,5]. In a study comparing the posterolateral approach and DAA, there were also better results with regard to function and pain for up to 3 months postoperatively with DAA [6].

The functional results in the present study show values comparable with those described in the literature, independently of the approach used. Perka et al. showed that the score increased from 38 points to 86 with a lateral approach. In the same study, patients who underwent surgery via the anterolateral approach had a mean of 84 points after the operation [24]. In the present study, excellent and good results could be achieved in 85.2%. Only 9.5% of the patients had moderate results, while 5.3% had poor results.

A meta-analysis by Moskal et al. confirmed similar results and showed that there are no significant differences in the complication rates between MIS and standard procedures [25].

In a cadaver study, implantation using a cemented technique via the DAA also showed no limitations in the quality of the bone cement, as well as correct stem positioning, thickness and distribution of the cement showed no significant differences to a transgluteal approach [26]. The present study also showed no incorrect positioning of components. The complication rate with cemented stems was 0%. The cases of aseptic stem loosening were due to a specific design problem in the cementless stem. In this stem, with metaphyseal fixation, it could be shown that there was a disproportion between the proximal and distal parts specifically with the larger components, so that distal locking occurred without osseous integration in the proximal part [27]. The design of this prosthesis has been changed, and a modified version is already in clinical use.

Higher complication rates have been reported particularly in association with initial implantations via the DAA. Intensive training is therefore indispensable. However, the same also applies to every new surgical technique [28].

Dislocation rates of 0.5–5.0% have been reported with primary THA [10,12,27]. With revision THA, the figures are 4.8–13% [7]. In 13–42% of the dislocations can only be reduced by surgery [10]. Dislocation is a complication that occurs in the first postoperative year in the great majority of cases; 75% of dislocations occur during the first 12 months, and 30–65% are in the first 3 months postoperatively [10]. The recurrence rate after dislocation is reported to be up to 65% [7]. Risk factors for dislocation have been examined in several studies. They include greater age, sex, prior surgery, and prior cognitive and neurological diseases. In addition, the surgical technique, implant characteristics, and the surgeon’s level of experience also influence the likelihood of dislocation [9]. In a study it was shown that the lateral approach led to dislocations in 3.1% of cases [29].

By contrast, dislocation rates of 6.9% and 4.6% have been reported with the posterolateral approach [17,30]. The dislocation rate in the present study was 0%. We would attribute this result to the approach selected, which spares the abductors and thus provides a high degree of stability in the hip joint, in combination with femoral head sizes ≥ 36 mm. But because of our retrospective design without a control group we cannot prove that especially the combination of DAA and large heads leads to this low dislocation rate. Maybe each factor itself is also able to reduce dislocation rates.A study could show that femoral head diameters of 36 mm or 32 mm reduce dislocation rates from 3.1% and 4.4% to as low as 0.8% in comparison with 28-mm heads [14-16]. Other studies have reported similar results and have emphasized the improved head–neck ratio in the prosthesis as the reason for the improvement [8-12]. In addition, the range of motion was improved with large heads as a result of reduced component-to-component impingement [8,10,31]. No dislocations were seen during the first 3 years even when high-risk factors such as advanced age, female sex and cup sizes > 60 mm were present [11,17,19,32].

The inlays used were all made of sequentially cross-linked X3™ polyethylene. Even in cup sizes of 46 mm, the use of a 36-mm head is possible. The mean linear wear rate with this tribologic combination was reported as 0.0004 mm/year in one study [21]. Highly cross-linked ultrahigh molecular weight polyethylenes [UHMWPEs] have lower wear rates – as little as 0.003 mm/year in vivo has been reported, in comparison with earlier-generation polyethylenes [0.051 mm/year] [33]. It has been shown that cross-linked polyethylene is associated with an 87% lower risk of osteolysis in comparison with conventional polyethylene [20]. Muratoglu et al. reported that the amount of wear is not dependent on the head size [34]. In a more recent study in 2009, however, it was shown that there was greater volumetric wear after 5 years in patients who had received heads larger than 36 mm [35]. During a 3-year follow-up period, no cases of wear-related osteolysis were noted radiographically in the present study, and no head decentering was observed on the digital radiographs. These results were independent of the head size selected.

In the initial phase, head sizes of 40 and 44 mm (n = 34) were used in the present group. During the subsequent course of the study, these sizes were no longer used, as the manufacturers ceased to produce them because of the low number of surgeons using these head sizes. Bartelt et al. reported that the rates of groin pain were 15% after large-head metal-on-metal THA and 18% after total hip resurfacing — much higher than the 7% noted with conventional-bearing THAs after a minimum of 1 year of follow-up. Potential factors involved were considered to be psoas impingement and higher activity levels [36]. No symptoms of impingement were noted in any of the 34 patients in the present study. But because of the midterm results with higher wear rates and the higher risk of groin pain because of psoas impingement it has to be discussed if heads greater then 36 mm are necessary. In our institution we do not use heads larger then 36 mm anymore.

## Conclusions

THA via the DAA, using head sizes ≥ 36 mm, is associated with good to very good functional results in the majority of cases. Higher complication rates are not observed. This combination of DAA and head size is particularly effective in countering the risk of dislocation. As these data are early results, however, regular radiographic check-up examinations are recommended in order to manage wear problems, which cannot be excluded during the longer-term course, particularly in younger and more active patients.

## Competing interests

The authors declare that they have no competing interests.

## Authors' contributions

SH: substantial contribution to conception and design, MS: substantial contribution to acquistion of data, GG: rivising the manuscript critically for content, HA: substantial statistical analysis and interpretation, RD: substantial contribution to acquisition of data, GH: rivising the the manuscript critically all authors approved the final version of the manuscript.

## Pre-publication history

The pre-publication history for this paper can be accessed here:

http://www.biomedcentral.com/1471-2474/15/80/prepub
